# Long-Term Disproportional TSH Hyposecretion in a Patient With Nonautoimmune Hyperthyroidism After Radioiodine Therapy

**DOI:** 10.1210/jcemcr/luad026

**Published:** 2023-03-22

**Authors:** Eijun Nishihara, Shuji Fukata, Akira Miyauchi, Takashi Akamizu

**Affiliations:** Department of Internal Medicine, Center for Excellence in Thyroid Care, Kuma Hospital, Kobe 650-0011, Japan; Department of Internal Medicine, Center for Excellence in Thyroid Care, Kuma Hospital, Kobe 650-0011, Japan; Department of Surgery, Center for Excellence in Thyroid Care, Kuma Hospital, Kobe 650-0011, Japan; Department of Internal Medicine, Center for Excellence in Thyroid Care, Kuma Hospital, Kobe 650-0011, Japan

**Keywords:** sporadic nonautoimmune hyperthyroidism, TSH hyposecretion, TSH receptor mutation, radioiodine therapy, large goiter

## Abstract

Nonautoimmune hyperthyroidism (NAH), caused by constitutively active mutants of the thyrotropin receptor (*TSHR*) gene, is recommended to be treated with total thyroidectomy followed by radioiodine administration. Herein, we present a 39-year-old woman with sporadic NAH caused by a TSHR-L512Q mutation. At the age of 20 years, she presented with a large goiter of 370 mL, treated with thiamazole, and opted for radioiodine therapy as outpatient management. Over the next 17 years, she underwent 6 treatments of 13 mCi radioiodine each. She did not experience a relapse of hyperthyroidism, and thiamazole was reduced and later withdrawn during the final radioiodine treatment. The patient's goiter significantly reduced to 18 mL, and thyroid function tests showed that free thyroxine and free triiodothyronine levels were below the lower limit of the reference ranges, while TSH remained within the reference range for 20 months. Along with an almost normal TSH response to thyrotropin-releasing hormone stimulation, no pituitary atrophy was observed on magnetic resonance imaging. Contrary to the recommended treatment, this case showed that fractionated radioiodine therapy alone is effective in controlling thyroid function and in reducing goiter size. Low TSH levels during treatment should not be assessed as subclinical hyperthyroidism or as risk of relapse.

## Presentation

**Figure 1.**
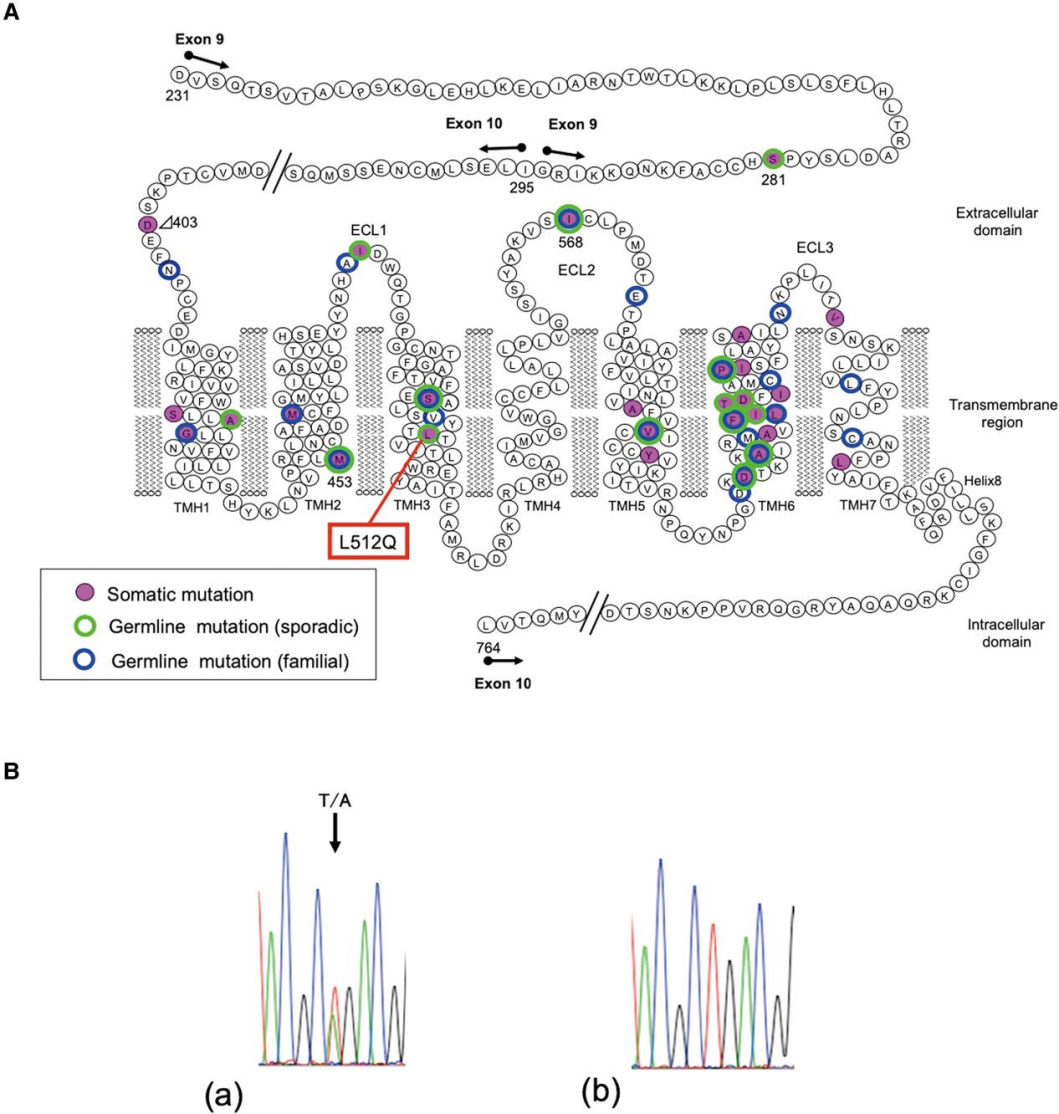
Constitutively active TSHR mutations in thyroid diseases. (A) The locations of TSHR mutations. (B) Sequencing analysis of *TSHR* exon 10 in genomic DNA. Heterozygous thymine to adenine transversion at position 1535 in patient (a) and none in control (b). Abbreviation: TSHR, thyrotropin receptor.

Nonautoimmune hyperthyroidism (NAH) is a rare disease caused by constitutively active mutants of the *TSHR* gene, which encodes the receptor for thyrotropin (TSH; thyroid-stimulating hormone). This disease is subdivided into 2 categories: familial NAH, due to autosomal dominant transmission, and sporadic NAH due to de novo mutations in the *TSHR* gene. To date, approximately 50 sporadic and familial cases have been reported (TSH receptor mutation database [https://tsh-receptor-mutation-database.org/]). Compared to familial cases, the clinical features of sporadic cases include an earlier onset and an increased severity of neonatal hyperthyroidism. The clinical severity of sporadic NAH is partially due to the higher activity of TSHR and is characterized by high compatibility with mutations in autonomous functioning nodules (Fig. 1A).

The management strategies for sporadic NAH are addressed by the guidelines of the European Thyroid Association based on case series [1]. It is imperative to control thyroid function using thiamazole and to prevent irreversible complications as soon as neonatal hyperthyroidism is identified. Subsequently, total thyroidectomy followed by radioiodine therapy is indispensable for preventing relapse of hyperthyroidism and goiter enlargement. In addition, long-term TSH suppression is likely, and the time for recovery of the feedback axis is unpredictable; hence, possible relapse of hyperthyroidism can go undetected [1]. However, the long-term clinical features of sporadic NAH without thyroidectomy are uncertain, especially in adulthood.

In this report, we described the 19-year follow-up of a patient with sporadic NAH (TSHR-L512Q) who underwent fractionated radioiodine therapy. The patient showed a marked decrease in goiter size without relapse of hyperthyroidism but experienced a central hypothyroid pattern with long-term disproportional TSH hyposecretion.

## Case Presentation

Ten days after birth, the patient was diagnosed with hyperthyroidism. As her condition was refractory to inorganic iodine therapy, thiamazole was initiated at the age of 5 months. Thereafter, she continuously received thiamazole treatment in combination with L-thyroxine (LT4). At the age of 20 years, she visited our hospital for consultation of her large goiter. Her thyroid function tests showed that serum TSH was 0.006 mIU/L (reference range, 0.30–5.00 mIU/L), free thyroxine (FT4) was 9.40 pmol/L (0.73 ng/dL) (reference range, 9.01–20.59 pmol/L [0.70–1.60 ng/dL]), and free triiodothyronine (FT3) was 17.66 pmol/L (11.50 pg/mL) (reference range, 2.61–5.68 pmol/L [1.70–3.70 pg/mL]) while taking 20 mg thiamazole and 50 μg LT4 daily. Tests for serum anti-thyroglobulin antibodies, anti-thyroid peroxidase antibodies, and TSH receptor antibodies were negative. Computed tomography (CT) of the neck revealed a diffuse goiter with a thyroid volume of 370 mL (normal, 5–20 mL).

### Diagnostic Assessment

For further investigation of congenital hyperthyroidism, sequence analysis of the patient's genomic DNA showed a heterozygous *TSHR* mutation (c.1535 T > A, p.L512Q) (Fig. 1A and 1B). This TSHR-L512Q mutant was previously reported to have constitutive activity [2] and was absent in her parents.

### Treatment

With sporadic NAH, total thyroidectomy and radioiodine therapy is recommended as radical therapy. However, the patient preferred the outpatient radioiodine therapy. In Japan, outpatient radioiodine dosage is limited to 500 MBq (13.5 mCi) per dose, and she underwent 6 treatments of 13 mCi radioiodine (cumulative dose of 78 mCi) over 17 years.

### Outcome and Follow-up

**Figure 2.**
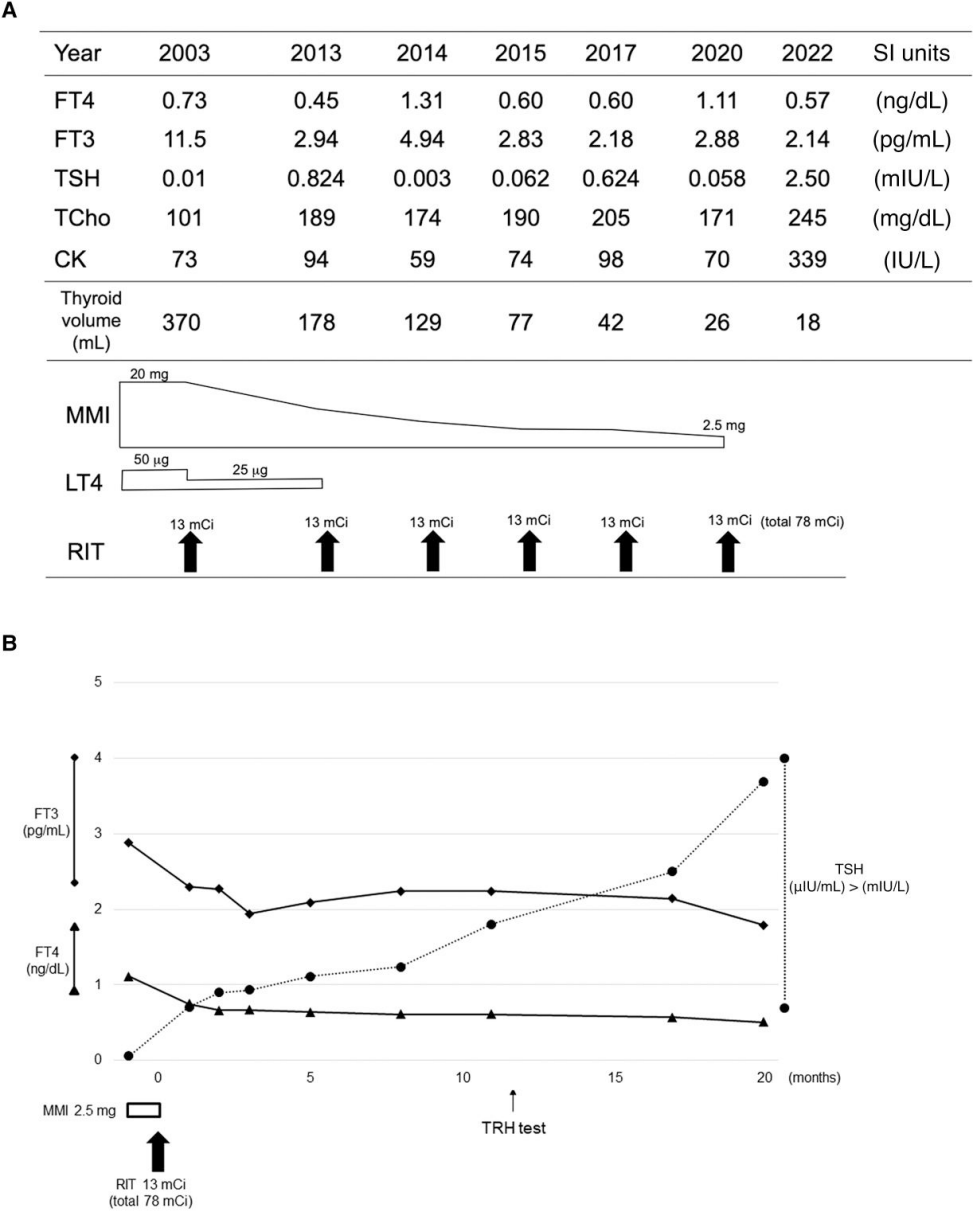
Sequential hormone and laboratory findings. (A) In the presence of MMI treatment with or without LT4 and fractionated RIT.(B) In the absence of MMI treatment after RIT (total 78 mCi). Reference ranges: TSH, 0.61–4.23 mIU/L; FT4, 11.58–21.88 pmol/L (0.90–1.70 ng/dL); FT3 3.53–6.14 pmol/L (2.30–4.00 pg/mL) Abbreviations: CK, creatinine kinase; MMI, thiamazole; LT4, levothyroxine; RIT, radioiodine therapy; TCho, total cholesterol.

**Figure 3.**
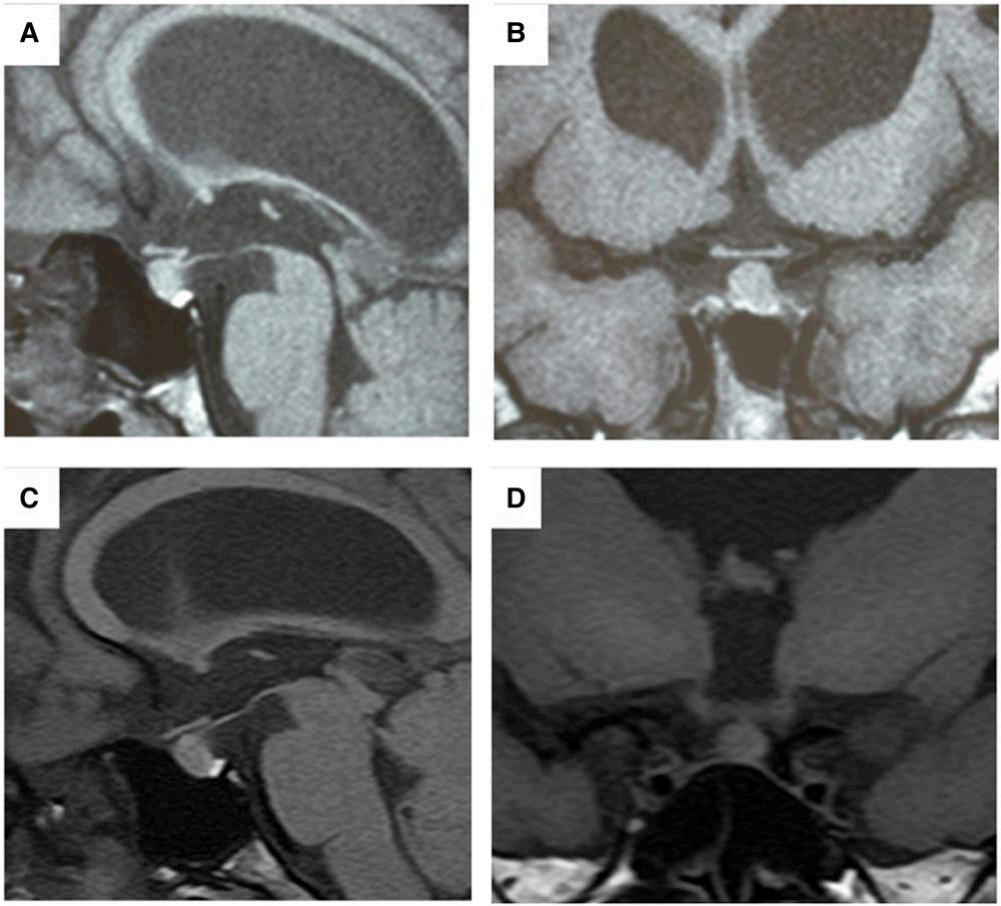
Brain magnetic resonance imaging (MRI) before radioiodine therapy. (A and B) before the final radioiodine therapy at 19 years of age; (C and D) after the final radioiodine therapy at 39 years of age. Sagittal (A and C) and coronal (B and D) images.

With the administration of radioiodine therapy, hyperthyroidism did not recur while thiamazole was reduced (Fig. 2A). Just before the final administration of radioiodine, thiamazole was withdrawn. Sequential changes in thyroid function tests showed that FT4 and FT3 levels were below the lower limit of the reference range, while TSH remained within the reference range for 20 months (Fig. 2B). The total cholesterol and creatinine kinase levels were elevated during this period, and the goiter size was significantly reduced to 18 mL (Fig. 2A). Brain magnetic resonance imaging (MRI) before radioiodine therapy and after 78 mCi administration showed hydrocephalus at the cerebral ventricles but no atrophy in the pituitary (Fig. 3). The thyrotropin-releasing hormone (TRH) test showed an almost normal response of TSH and prolactin (PRL), with a peak at 30 minutes after administration (8.94 mIU/L and 4.48 nmol/L [103.0 ng/mL], respectively); however, at 120 minutes, the ratio of total T3 to that of prior administration was slightly low (112%; normal, > 120%) (Table 1). Tests for serum anti-thyroglobulin antibodies, anti-thyroid peroxidase antibodies, and TSH receptor antibodies were continuously negative. There was no evidence of interferences for TSH measurement by TSH dilution test and polyethylene glycol precipitation test or no history about biotin ingestion. There were no differences in hormone levels as measured using other assay kits.

**Table 1.** Thyrotropin-releasing hormone test

Time (minutes)	0	30	60	90	120
TSH (mIU/L)	1.58	8.94	6.21	4.31	3.51
T3 (nmol/L [ng/mL])	1.39 (0.91)	NT	NT	NT	1.57 (1.02)
PRL (nmol/L [ng/mL])	1.18 (27.2)	4.48 (103.0)	2.17 (49.9)	1.60 (36.7)	1.22 (28.0)

Abbreviations: NT, not tested; PRL, prolactin; T3, triiodothyronine; TSH, thyroid-stimulating hormone.

## Discussion

We described the 19-year follow-up of a patient with sporadic NAH (TSHR-L512Q) who achieved a marked decrease in size of a large goiter after radioiodine therapy and, subsequently, presented with disproportional TSH hyposecretion. Previously, we reported that this patient developed irreversible complications, such as bone abnormalities, hydrocephalus, and intellectual disability, due to poor control of hyperthyroidism during the neonatal period [3]. After diagnosing the patient with NAH at the age of 20 years, the goal of treatment was to control the enlarged goiter and hyperthyroidism that was refractory to thiamazole. The European Thyroid Association guidelines strongly recommend complete ablation of thyroid tissue by total thyroidectomy, followed by radioiodine therapy to avoid relapse of hyperthyroidism and goiter [1]. In this patient, fractionated radioiodine therapy led to remission of hyperthyroidism without relapse and reduced the goiter volume from 370 to 18 mL during a long follow-up period (Fig. 2A). Previously, we applied repeated low-dose radioiodine therapy to a patient presenting with hyperfunctioning metastases secondary to follicular thyroid carcinoma; and her severe hyperthyroidism successfully improved with reduced uptake in metastatic lesions [4]. Both these diseases are characterized by absence of TSH receptor antibodies, which is advantageous in preventing hyperthyroid exacerbation after radioiodine therapy; however, patients with Graves hyperthyroidism often experience exacerbation due to elevated levels of TSH receptor antibodies. Therefore, repeated radioiodine therapy is a reasonable option for older children or adult patients, but not likely for younger patients with NAH, in whom total thyroidectomy is not possible.

After radioiodine therapy and in the absence of thiamazole, the patient exhibited a central hypothyroid pattern with lower levels of FT4 and FT3 but normal TSH levels for 20 months (Fig. 2B). There were no gross morphological changes in the pituitary before or after radioiodine therapy (Fig. 3), and the response of TSH production after TRH stimulation was preserved (Table 1). However, elevation of cholesterol and creatine kinase levels (Fig. 2A) and mild impairment of T3 elevation after TRH stimulation (Table 1) indicated changes to hypothyroidism in the peripheral tissues. Although a transient shift to the central hypothyroid phase can be detected in 90% of patients with Graves hyperthyroidism after radioiodine therapy, it persists for an average of 24.7 ± 2.4 days (range, 14–47 days) until the recovery of the hypothalamic-pituitary-thyroid (HPT) axis; 89% of these patients develop primary hypothyroidism with TSH elevation above the upper limit of the reference range [5]. It has also been proposed that TSH suppression persists after radioiodine therapy because of pituitary atrophy, and the lag time in TSH recovery corresponds to the time needed for atrophied thyrotropes to regain function [6]. These findings suggest that the curiously low TSH status in our case was neither transient after radioiodine therapy nor caused by pituitary atrophy and that other mechanisms may be involved.

Central hypothyroidism can be detected in newborns of mothers with uncontrolled gestational hyperthyroidism [7]. In these cases, the increased transplacental transfer of maternal T4 is thought to disturb maturation and regulation of the HPT axis of the fetus. Since the TSH suppressive state persists due to untreated congenital hyperthyroidism in the NAH-affected fetus, the TSH setpoint might be similarly impaired. Otherwise, the constitutive activation of TSHRs localized on follicular stellate cells in the pituitary might be involved in the inadequate TSH production via a mechanism known as autoregulatory ultrashort-loop feedback control [8]. TSH dysregulation with a central hypothyroidism pattern was observed in 2 other patients with NAH after radioiodine therapy [9, 10]. The location and activity of these mutated TSHRs vary. The exact mechanism of disproportional TSH hyposecretion remains unclear, but it is more likely to be evident in patients with NAH who underwent radioiodine therapy during their clinical courses.

In conclusion, fractionated radioiodine therapy without total thyroidectomy is effective in some adult patients with NAH. Unexpected outcomes show that this treatment may adequately control thyroid function without relapse of hyperthyroidism, as well as reduce enlarged goiters. During treatment, low TSH levels should be considered neither subclinical hyperthyroidism nor risk of relapse.

## Learning Points

- Fractionated radioiodine therapy without total thyroidectomy is effective for remission of hyperthyroidism without relapse and reduced the goiter volume in some adult patients with NAH.
- After radioiodine therapy, the patient exhibited a long-term central hypothyroid pattern with disproportional TSH hyposecretion.
- Low TSH levels in patients with NAH should be considered neither subclinical hyperthyroidism nor risk of relapse.

## Data Availability

Original data generated and analyzed during this study are included in this published article.

## Abbreviations

FT3: free triiodothyronine
FT4: free thyroxine
LT4: levothyroxine
NAH: nonautoimmune hyperthyroidism
TRH: thyrotropin-releasing hormone
TSH: thyrotropin (thyroid-stimulating hormone)
TSHR: thyroid-stimulating hormone receptor
